# A counterpoint to “training the translational workforce: expanding beyond translational research to include translational science”

**DOI:** 10.1017/cts.2020.32

**Published:** 2020-04-06

**Authors:** Paul R. Marantz, Marla J. Keller, Emma A. Meagher

**Affiliations:** 1 Albert Einstein College of Medicine and Montefiore Medical Center, Bronx, NY, USA; 2 Perelman School of Medicine, University of Pennsylvania, Philadelphia, PA, USA

**Keywords:** Discipline, education, training, translational research, translational science

We were intrigued to read our colleagues’ thoughtful consideration of the recently proposed distinction between “translational research” and “translational science.” Drs. Tsevat and Smyth conclude that this distinction has important implications for training the next generation of investigators and that Clinical and Translational Science Award (CTSA)-supported education and career development programs should consider developing new competencies and learning modules to support this “new discipline of translational science” [1]. We respectfully disagree with this conclusion and a similar sentiment reported in two previous manuscripts [2, 3]. Indeed, we question whether the distinction is actually meaningful and would like to suggest that this hypergranular fragmentation of our discipline may be ill-advised and possibly harmful.

First, is translational science actually a “new discipline”? This involves two component questions: is it a “discipline,” and is it “new”? The word *discipline* is most simply (and inclusively) defined as “a field of study” [4]. Certainly, this gives quite a bit of wiggle room in determining what is and what is not a discipline, but “clinical and translational science” was defined as such by Dr. Elias Zerhouni in a frequently cited and widely read “Sounding Board” piece, published in 2005 in the *New England Journal of Medicine* [5] as he unveiled the National Institutes of Health (NIH)’s new CTSA program. He described it as “… an emerging discipline that encompasses both the acquisition of new knowledge about health and disease prevention, preemption, and treatment and the methodologic research necessary to develop or improve research tools.” Clearly, this last clause already incorporates the definition of “translational science” suggested in our colleagues’ commentary piece into the original NIH definition of “translational research.” Even if we accept this designation as a “discipline,” whether it can be considered “new” 15 years later is similarly open to interpretation.

Beyond these semantics (discipline? new?), it is worth thinking about the underlying meaning and implications of this designation. In academia, we sometimes equate “discipline” with “department”; indeed, in the lead-up to the first CTSA request for applications (RFA) [6], the push appeared to be toward creating new departments of Clinical and Translational Science. In the event, however, the RFA allowed for the creation of “an academic home, which can be a center, department, or Institute (C/D/I),” and the academic medical community overwhelmingly opted for the Center or Institute approach.

Let us look at the first published definition of “translational science”: “National Center for Advancing Translational Sciences (NCATS) defines it as the field of investigation which seeks to understand the scientific and operational principles underlying each step of the translational process” [2]. If this is meant to represent a scholarly “discipline,” this definition makes it unlike any other we are aware of. It would be as if a discipline of “biochemistry science” were created in parallel to “biochemistry,” with the former focused entirely on trying to understand how biochemical research is conducted.

Tsevat and Smyth’s argument for the need to develop new competencies for training “translational scientists” (to be distinct from those previously developed for “translational researchers”) includes the following text:

A recent commentary proposed a set of 7 characteristics fundamental to translational scientists: (1) boundary crosser; (2) domain expert; (3) team player; (4) process innovator; (5) skilled communicator; (6) systems thinker; and (7) rigorous researcher [2]. This vision of translational scientists contrasts with that of translational researchers, who more often focus on a disease or content-specific area of investigation and/or adopt a particular phenotype (e.g. clinical investigator or data scientist), although phenotypes can certainly evolve across a career.

While any given translational researcher typically does focus his or her studies on a particular disease or content area, we believe these same seven listed characteristics equally apply and are relevant to the successful outcome of all of our translational research trainees. Of course, some of those characteristics will be of varying importance for translational researcher “phenotypes”: for example, a “systems thinker” as defined in that paper is critical for someone doing patient-centered outcomes research while less so for someone doing bench-to-bedside studies, further supporting our argument that we really do not need additional fragmenting or creating another unnecessary competing and confusing discipline. As depicted in the Venn diagram (Fig. 1 in [1]), the listed competencies of the “new discipline” fit under the long-standing and accepted construct of “translational research.”

Beyond the personal preferences of nomenclatures and differentiators, it is the utilitarian question we come back to: what benefit or harm might we expect if there was an appetite to create this “new discipline”? Even if one were convinced that this framework is helpful in some way, it is clear that existing CTSA-driven educational programs currently address these competencies, albeit using different vernacular than the terms put forth previously [2, 3] and by our colleagues (team science, leadership, project management among others). In addition, any potential for reallocation of current, suboptimal levels of funding for training our scholars and trainees to this new discipline could be harmful.

The CTSA program has appropriately and successfully pushed us toward breaking down long-standing institutional and disciplinary boundaries as we conduct clinical and translational research. We recognize that the techniques of “process engineering,” as more traditionally exemplified by our engineering colleagues, are relevant to this undertaking, but not to the degree that it requires a new discipline. In fact, it could be argued that each CTSA hub is already studying the process of translation by participation in the Trial Innovation Network (TIN). The vision of the TIN is to accelerate the translation of novel interventions into therapies. The goal is to not only execute trials better and faster but also to study and understand the process of conducting clinical trials in order to promote operational innovation.

We think spinning off “translational science” creates a new silo that no one needs. Let us work together with scientists from all the relevant disciplines to plan, conduct, analyze, interpret, disseminate, and apply the best possible studies to improve the health of all.
